# Combining prostate-specific antigen density with prostate imaging reporting and data system score version 2.1 to improve detection of clinically significant prostate cancer: A retrospective study

**DOI:** 10.3389/fonc.2022.992032

**Published:** 2022-09-23

**Authors:** Yin Lei, Tian Jie Li, Peng Gu, Yu kun Yang, Lei Zhao, Chao Gao, Juan Hu, Xiao Dong Liu

**Affiliations:** ^1^ Department of Urology, The First People’s Hospital of Shuangliu District, Chengdu, China; ^2^ School of Clinical Medicine, Tsinghua University, Beijing, China; ^3^ Department of Urology, The First Affiliated Hospital of Kunming Medical University, Kunming, China; ^4^ Medical school, University of Electronic Science and Technology of China, Chengdu, China; ^5^ Medical Imaging Department, The First Affiliated Hospital of Kunming Medical University, Kunming, China

**Keywords:** prostate biopsy, prostate cancer, prostate imaging reporting and data system score, prostate-specific antigen density (PSAD), clinically significant prostate cancer (csPCa)

## Abstract

Globally, Prostate cancer (PCa) is the second most common cancer in the male population worldwide, but clinically significant prostate cancer (CSPCa) is more aggressive and causes to more deaths. The authors aimed to construct the risk category based on Prostate Imaging Reporting and Data System score version 2.1 (PI-RADS v2.1) in combination with Prostate-Specific Antigen Density (PSAD) to improve CSPCa detection and avoid unnecessary biopsy. Univariate and multivariate logistic regression and receiver-operating characteristic (ROC) curves were performed to compare the efficacy of the different predictors. The results revealed that PI-RADS v2.1 score and PSAD were independent predictors for CSPCa. Moreover, the combined factor shows a significantly higher predictive value than each single variable for the diagnosis of CSPCa. According to the risk stratification model constructed based on PI-RADS v2.1 score and PSAD, patients with PI-RADS v2.1 score of ≤2, or PI-RADS V2.1 score of 3 and PSA density of <0.15 ng/mL^2^, can avoid unnecessary of prostate biopsy and does not miss clinically significant prostate cancer.

## Introduction

Prostate cancer (PCa) accounts for 13.5% of all cancer cases and 6.7% of all cancer deaths among males worldwide, ranking second and sixth for cancer incidence and mortality among men respectively (1). Most prostate cancers are not aggressive and represent little or no damage to the patient’s health or life expectancy, despite the disease’s high occurrence. Many will not be diagnosed with prostate cancer until an autopsy or screening is performed. Although there is no standardized definition of clinically significant prostate cancer, the disease has become more aggressive. However, clinically significant prostate cancer is an aggressive, fatal disease that causes death in some men; definite treatment is required. Prostate-specific antigen (PSA) testing is crucial for the diagnosis of prostate cancer, which has led to a decrease in disease-specific mortality and advanced disease during the previous two decades (2). Regrettably, PSA testing alone increased the detection of many clinically insignificant prostate cancer, which usually progress indolently and does not need any clinical intervention (3). Therefore, It is imperative to establish a non-invasive method to prevent over-diagnosis and eliminate unnecessary biopsies, while simultaneously identifying clinically significant prostate cancer as early as possible.

Multiparametric Magnetic Resonance Imaging (mpMRI) that combines T2-weighted imaging (T2WI) with functional pulse sequences such as dynamic contrast-enhanced (DCE) and/or diffusion-weighted imaging (DWI) imaging has demonstrated high application value in PCa diagnosis, local staging, and active surveillance. PI-RADS v2.1 was recommended to assess the likelihood of a clinically significant cancer of any lesion based on mpMRI in the prostate using a 5-level subjective score (4). A meta-analysis found that the median mpMRI negative predictive value (NPV) was 82.4% (IQR, 69.0–92.4%) for overall cancer and 88.1% (IQR, 85.7–92.3) for CSPCa (5). PRECISION trial (6) and PROMIS trial (7) demonstrated that the use of mpMRI to triage men prior to prostate biopsy could allow a quarter of men to avoid a primary biopsy and reduce the number of clinically insignificant cancer missed. The significance of PI-RADS point 3 for the diagnosis of PCa and CSPCa has, however, varied considerably between investigations (8, 9). The suspicious lesion concerns the presence of clinically relevant cancer was assigned PI-RADS point 3 per the standards. As a result, managing unclear or ambiguous PI-RADS 3 lesions has become difficult for doctors. To overcome these shortcomings and increase the consistency of physician assessments, the PI-RADS Steering Committee has revised PI-RADS v2 based on consensus (PI-RADS v2.1).Previous studies have validated the diagnostic performance of PI-RADS v2.0 score combined with PSAD in the detection of CSPCa. However, due to inconsistent methodology across different studies, heterogeneous outcomes were observed. In some studies, PI-RADS v2.0 scores were assessed based on 1.5T MRI machine, while the others were based on 3.0T machine (10), In addition, some studies apply MR protocol that only consists of T2WI and DWI, which is called bi-parametric MRI(bpMRI) does not precisely meet the requirements of PI-RADS v2.0 system (10). In contrast, the majority of studies lack follow-up information for patients whose biopsies were negative (11, 12). Consequently, the purpose of the current study is to further validate the performance of PI-RADS v2.1 score combined with PSAD in the detection of CSPCa, using a more accurate PI-RADS v2.1 score based on a 3.0T machine that includes T2WI, DWI, and DCE.

## Materials and methods

### Patients selection

We retrospectively reviewed a cohort including 422 patients who underwent mpMRI prior prostate biopsy and underwent their first prostate biopsy between January 2016 and January 2019 at the First Affiliated Hospital of Kunming Medical University. Inclusion criteria: 1. Patients with suspected prostate cancer found by a rectal exam, PSA, TRUS, MRI; 2. Patients willing to undergo prostate puncture biopsy. The exclusive criteria were as follows: 1) lack of any T2WI, DWI and DCE; 2) lack of histopathological results or clinical information, including age, PSA, fPSA and MRI-measured prostate volume; 3) the previous history of prostate surgery; 4) received 5α reductase inhibitors; 5) lost to follow-up.

### MRI

All mpMRI scans of the prostate were performed with 3.0T MR scanner (Achieva, Philips/Discovery MR W750, GE), which involved axial T2WI, DWI, and DCE. The Apparent Diffusion Coefficient (ADC) map was automatically calculated. And the locations of these axial sequences were exactly matched. PI-RADS score of each case was graded separately according to the PI-RADS v2.1 criteria by two independent radiologists (R1, R2) blinded to the clinical information and pathological outcomes. If scores were inconsistent, the final PI-RADS scores were determined through a discussion between two radiologists. The volume of the prostate was measured according to the PI-RADS v2.1 criteria based on mpMRI: ([maximum anteroposterior {AP} diameter] X [maximum transverse diameter] X [maximum longitudinal diameter] X 0.52), the maximum AP and longitudinal diameters are placed on the midsagittal T2W image, while the maximum transverse diameter is placed on the axial T2W image. And the TNM staging of prostate cancer was determined mainly based on mpMRI by the radiologist(R2).

### Prostate biopsy and pathological analysis

The indications of prostate biopsy and repeated biopsy were performed in accordance with the Chinese Urology Association Guidelines and European Association of Urology Guidelines. In all patients, 12-core systematic transrectal ultrasound-guided prostate biopsies were performed by urinary specialists with more than 10 years of experience, and two cognitive fusion-targeted biopsy cores were added for each lesion based on mpMRI findings.

The final pathological results of this study are subject to biopsy and follow-up results. Patients who received negative results in their initial biopsies were followed up on, which included repeat biopsy results, surgical therapy results, MRI results, and PSA results. Clinically significant prostate cancer was defined as Gleason score≥3+4 or ≥T3 staging (extracapsular extension). Clinically insignificant prostate cancer was defined as Gleason score<3+4 or ≤T2 staging.

### Statistical analysis

For normally and non-normally distributed data, the mean (standard deviation [SD]) and the median (interquartile range [IQR]) will be used. To assess between-group differences in normally and non-normally distributed data, the Student t-test and Mann-Whitney U-test were used. Categorical variables were represented as percentages, and chi-square test was used to assess between-group differences. The area under the curve (AUC) was used to assess the accuracy of the receiver operating curves (ROC) for factors evaluated for the risk of PCa and CSPCa. PSAD was divided into four subgroups based on the appropriate PSAD cut-off points for detecting PCa and CSPCa and recognizing outliers, and the risk category for CSPCa was constructed using PI-RADS V2.1 scores and PSA subgroups.

*P* value less than 0.05 was considered to indicate a statistically significant. SPSS software was used to conduct all analyses (Version 20.0. IBM).

## Results

### Patients data

The Profiles of 422 patients were analyzed. As stated in **Table 1**, the mean age was 68.50 ± 7.44 years. The median values for [interquartile range (IQR)] tPSA, f/tPSA, PV and PSAD were, 14.20(8.24~37.18) ng/mL, 0.15(0.11~0.22), 56.69(37.70~77.08) ml, and 0.27(0.15~0.76) ng/ml^2^, respectively. The number of PI-RADS V2.1 score 1-2, 3, 4, 5 were 167, 53, 67, 135, respectively.

**Table 1:** Patients’ characteristics.

Variables	Value
Median (IQR)	
tPSA(ng/ml)	14.21(8.25~37.90)
f/tPSA	0.15(0.11~0.21)
PV (ml)	54.66(37.31~74.98)
PSAD (ng/ml^2^)	0.27(0.15~0.77)
Mean ± SD	
Age (years)	68.49 ± 7.47
N (%)	
PI-RADS v2 score	
1-2	167(39.6%)
** 3**	**53(12.6%)**
** 4**	**67(15.8%)**
** 5**	**135(32.0%)**

### Pathological outcomes

**Figure 1:**
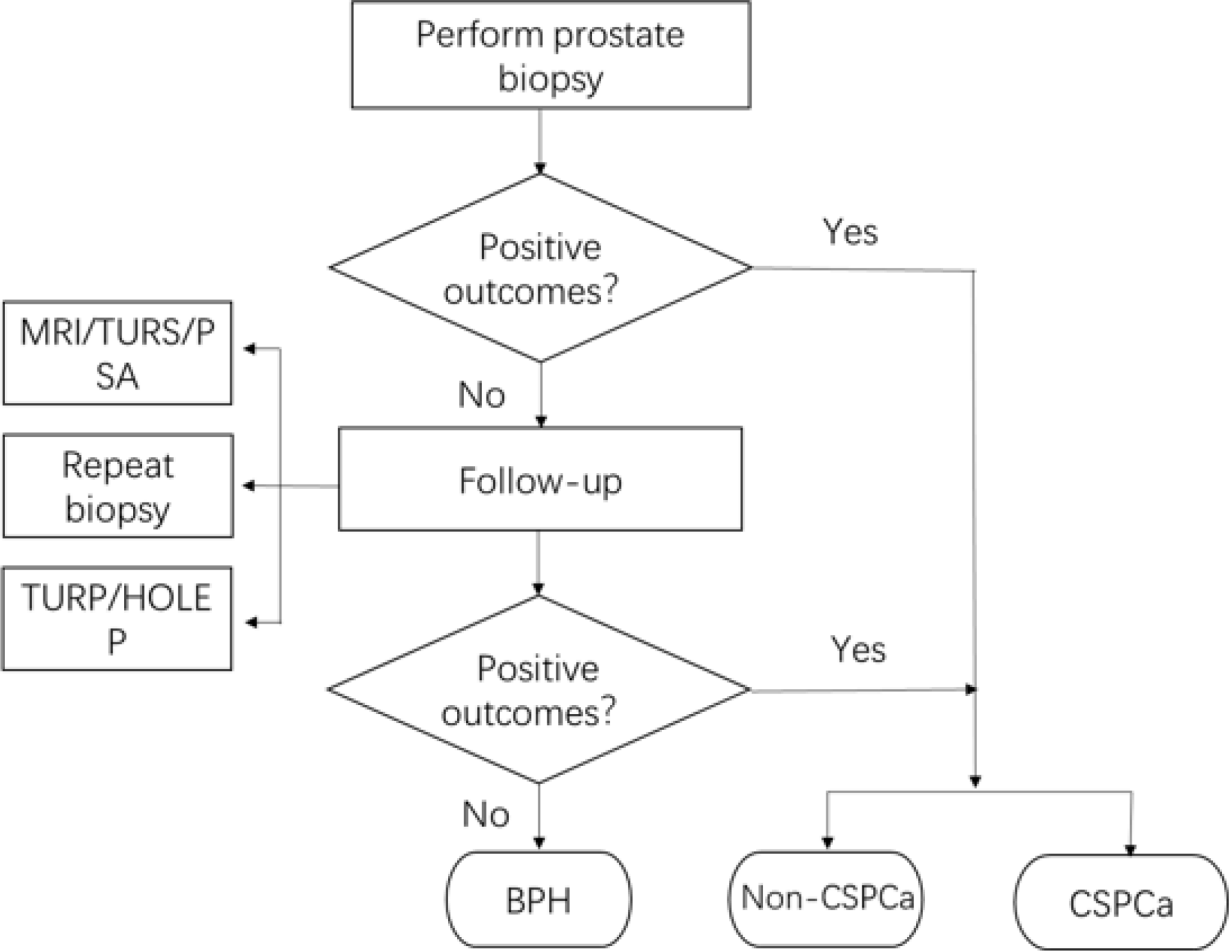
Pathological outcomes flow chart.

The flow chart of pathological outcomes is depicted in **Figure 1**. 194 patients were confirmed with positive outcomes from the initial biopsy, of which 149 patients were diagnosed with CSPCa. In the meantime, 228 patients were diagnosed with BPH in their initial biopsy and would be followed up; of which 29 patients underwent a repeat biopsy and 2 of them were diagnosed with PCa, including 1 CSPCa; 123 patients underwent TURP or HOLEP, and 2 patients of them were diagnosed with CISPCa; and 11 patients who underwent both biopsy and TURP were diagnosed with BPH. Regular monitoring of PSA, MRI, and transrectal ultrasound, if needed, demonstrated the absence of disease development for 85 patients. Finally, the pathological results for benign prostate hyperplasia (BPH), PCa, and CSPCa were 221(53.1%), 201 (46.9%), and 150(35.5%), respectively.

### Group analysis

First of all, we divide all patients into groups based on the following criteria, 1) BPH group and PCa group according to their pathological results, 2) CSPCa group and non-CSPCa group (including CISPCa and BPH patients) according to whether the pathological outcome is CSPCa. Then we analyzed the differences of risk factors by groups described above, 1) BPH group and PCa group, 2) CSPCa group and non-CSPCa group.

As shown in **Table 2**, the BPH group and the PCa group contained 224 and 198 patients respectively. While the CSPCa group and non-CSPCa group contained 150 and 272 patients respectively. Intriguingly, we discovered that biopsy results were significantly correlated with age, PSA, PV, PSAD, and MRI findings (all p < 0.05, **Table 2**) between CSPCa and non-CSPCa group, as well as BPH and PCa group.

**Table 2:** Univariate analysis of the Clinical Characteristics in the different groups.

Variables	Group 1			Group 2		*P* value
	PCa	BPH	*P* value	CSPCa	Non-CSPCa	
patients	198(46.9%)	224(53.1%)	–	150(35.5%)	172(64.5%)	–
**Age**	69.85 ± 7.35	67.29 ± 7.38	*P*<0.001^1)^	69.45 ± 7.36	67.69 ± 7.49	*P*<0.001^1)^
**tPSA (ng/ml)**	34.85(12.37~100.00)	10.36(6.85~16.07)	*P*<0.001^2)^	71.85(17.88~100.00)	10.76(7.18~16.72)	*P*<0.001^2)^
f/tPSA	0.15(0.10~0.24)	0.15(0.11~0.19)	*P*=0.54^2)^	0.16(0.09~0.26)	0.15(0.11~0.20)	*P*=0.527^2)^
**PV (ml)**	48.40(32.31~66.97)	60.52(44.03~85.85)	*P*=0.003^1)^	49.29(32.31~66.97)	57.74(42.56~85.55)	*P*=0.036^1)^
**PSAD (ng/ml2)**	0.76(0.29~1.46)	0.17(0.12~0.28)	*P*<0.001^2)^	1.01(0.43~1.71)	0.18(0.12~0.29)	*P*<0.001^2)^
**MRI (%)**			*P*<0.001^3)^			*P*<0.0013)
positive	187	68		150	14	
**negative**	11	156		0	34	

PCa, prostate cancer; CSPCa, clinically significant prostate cancer; PSA, prostate-specific antigen; f/tPSA, the ratio of free to total prostate specific antigen; PV, prostate volume; PSAD PSA density.

### Efficiency of risk factors in the diagnosis of PCa and CSPCa

Then, we aimed to identify PCa and CSPCa-associated risk variables. As shown in **Table 3**, the AUC values of PI-RADS v2.1 score and PSAD were 0.91 and 0.84, 0.95 and 0.89 for PCa and CSPCa, respectively, which was greater than all other factors (P<0.05). Then we determined that PI-RADS v2.1 score 4 as the cut-off point for distinguishing PCa and CSPCa, and 0.38 and 0.65 as the cut-off of PSAD for diagnosing PCa and CSPCa, respectively. Finally, the cutoff for PI-RADS v2.1 score and PSAD were selected for the prediction models, which were constructed for discriminating PCa and CSPCa. Consequently, the AUC of the prediction model consists of PI-RADS v2.1 score and PSAD was higher for PCa and CSPCa in comparison to PI-RADS v2.1 score and PSAD alone (**Table 3**).

**Table 3:** Diagnostic performance of risk factors for PCa and CSPCa.

Variable	PCa						CSPCa					
	AUC	SEN	SPE	PPV	NPV	Cut-off	AUC	SEN	SPE	PPV	NPV	Cut-off
PI-RADS v2 score	0.91	85.4	85.3	83.7	86.8	4	0.95	97.3	79.4	72.3	98.2	4
PSAD	0.84	68.2	87.5	56.4	80.6	0.38	0.89	70.0	94.9	88.2	85.1	0.65
PSA	0.79	57.1	89.3	82.5	70.2	24.0	0.85	68.0	91.5	91.6	83.8	31.2
f/tPSA	0.52	16.7	98.7	91.7	57.3	0.33	0.52	20.0	97.8	83.3	68.9	0.33
PV	0.62	54.0	65.6	58.1	61.8	50.1	0.59	42.7	73.2	46.7	69.8	43.4
age	0.59	48.0	66.5	55.9	59.1	70	0.55	47.3	63.6	41.8	68.6	70
PI-RADS +PSAD	0.93	90.4	80.8	80.6	90.5	–	0.97	92.7	87.9	80.8	95.6	–

PCa, prostate cancer; CSPCa, clinically significant prostate cancer; CISPCa, clinically insignificant prostate cancer; PSA, prostate-specific antigen; f/tPSA, the ratio of free to total prostate specific antigen; PV, prostate volume; PSAD, PSA density; AUC, area under of curve; SEN, sensitivity; SPE, specificity; PPV, positive predictive value; NPV, negative predictive value.

### Construction of a multivariate risk category to predict CSPCa

For further analysis, we divided PSAD into four subgroups based on the study-confirmed cutoff point(0.38, 0.65) and widely accepted threshold (0.15) (**Table 6**). The examination of multivariate logistic regression indicated that PI‐RADS v2 score and PSAD were independent predictors of CSPCa. Further analysis revealed no significant difference was observed for CSPCa for PI-RADS v2.1 score 2 and 3 (**Table 4**).

**Table 4:** The multivariate logistic regression analysis of PI‐RADS v2 score and PSAD for CSPCa.

Variables	OR	95%CI	*P* value
PSAD (ng/ml^2^)			–
<0.15	–	–	P<0.001
~0.38	0.060	0.016~0.223	P<0.001
~0.65	0.126	0.050~0.321	P<0.001
≥0.65	0.320	0.104~0.983	P<0.001
PI-RADS V2 score			
2	–	–	–
3	0	0	P=0.994
4	0.029	0.009~0.100	P<0.001
5	0.148	0.065~0.336	P<0.001

PIRADS v2 prostate imaging-reporting and data system version 2; PSAD, PSA density; OR, odds ratio; CI, confidence interval.

Next, we attempted to validate our findings about CSPCa prediction. As indicated in **Table 5**, patients with a PI-RADS v2.1 score of 2 were negative for CSPCa, while 53 patients with a PI-RADS v2.1 score of 3 were diagnosed with CSPCa. Moreover, only 2 out of 108 individuals with a PSAD of 0.15 ng/mL2 were proven to have CSPCa. Therefore, we developed a risk category for CSPCa based on the combined PI-RADS v2.1 score of PSAD subgroups.

**Table 5:** Detection of PSAD subgroups and PI-RADS v2 score for PCa and CSPCa.

Outcomes	PSAD subgroups				PI-RADS v2 score			
	<0.15	~0.38	~0.65	≥0.65	1-2	3	4	5
patients	108	150	44	120	167	53	67	135
PCa(n, %)	21(20.9)	42(28.0)	26(59.1)	109(90.8)	11(6.6)	18(34.0)	44(65.7)	125(92.6)
CSPCa(n, %)	7(3.6)	25(16.7)	13(29.5)	105(87.5)	0(0)	4(7.5)	26(38.8)	120(88.9)

PCa, prostate cancer; CSPCa, clinically significant prostate cancer; PIRADS v2, prostate imaging-reporting and data system version 2; PSAD, PSA density.

As demonstrated in **Table 6**, we confirmed that PI‐RADS v2 score of ≥4, or a PI‐RADS v2 score of 3, and a PSAD≥0.65 ng/mL^2^ (red zones) as the high-risk group, with the highest CSPCa detection rate (72.1%). In contrast, a PI‐RADS v2 score of 2, or a PI‐RADS v2 score of 3 with PSAD of ≤0.15 ng/mL^2^ (green zones), were assigned as the low‐risk group in which no CSPCa was detected. Others (blue zones) with a 10.3% detection rate for CSPCa were assigned as the moderate‐risk group. The detection rates for PCa in patients assigned with low‐, moderate‐, and high‐risk prostate cancer were 9.0%, 37.9%, and 83.3%, respectively.

**Table 6:** Risk category of CSPCa.

		PI-RADS v2 score			
		1-2	3	4	5
PSAD	<0.15	0 (0/72)	0 (0/22)	28.6% (2/7)	71.4% (5/7)
	~0.38	0 (0/77)	15% (3/20)	30.3% (10/33)	60.0% (12/20)
	~0.65	0 (0/10)	0 (0/9)	25.0% (3/12)	76.9% (10/13)
	≥0.65	0 (0/8)	50.0% (1/2)	73.3% (11/15)	97.9% (93/95)

Red, green and blue zones indicate high‐, moderate‐ and low‐risk groups, respectively. The detection rates for PCa in patients assigned with low‐, moderate‐, and high‐risk prostate cancer were 9.0%, 37.9% and 83.3%, respectively.

## Discussion

The previous report in China (44%, 6123/13904), as published by 33 member hospitals of the Chinese Prostate Cancer Consortium (CPCC) (13). We enrolled 422 patients in our trial for a minimum of 14 months follow-up, and we discovered the same outcome. 53.1 percent (224/422) of patients were diagnosed with benign lesions (prostatic hyperplasia, prostatitis, etc.), while 46.9 percent (198/422) were diagnosed with PCa.

Since the release of PI-RADS V2 based on mpMRI, PI-RADS v2.0 has been widely recognized in radiology and urology, as well as clinical practice. Its clinical value and practicability have been extensively validated. Current studies have also shown that PI-RADS V2.1 has excellent performance in predicting PCa, particularly for CSPCa (14–17), with an even higher accuracy over systematic TRUS biopsies for PCa diagnosis (6, 7, 18). In this study, PI-RADS v2.1 score was an independent predictor for PCa with excellent diagnostic performance, the AUC was 0.9108 with PI-RADS v2.1 score 4 as the cut-off. The sensitivity, specificity, PPV and NPV were 85.4%, 85.3%, 83.7% and 86.8%, respectively.

For CSPCa, We defined clinically significant prostate cancer as Gleason score≥3+4 or ≥T3 staging (extracapsular extension). PI-RADS v2.1 has improved diagnostic accuracy. The AUC was 0.95 with PI-RADS v2.1 score 4 as the cut-off, and NPV was up to 98.2%. A high NPV can help minimize unnecessary prostate biopsies and their associated problems.

Although the PI-RADS score had an advantage in predicting CsPCa in this study, PI-RADS V2.1 of 4 score has s NPV up to 98.2% for CSPCa, If the biopsy was carried on a PI-RADS score = 3, 7.5% (4/53) of CsPCa patients would be omitted; however, if set at a PI-RADS score≥3, 19.2% (49/255) of patients would receive an unnecessary biopsy. Overtreatment of inactive prostate cancer diminishes the quality of life, but the delayed treatment of more aggressive CSPCa increases the incidence of metastatic illness and mortality. Therefore, a decision to puncture the prostate based exclusively on PI-RADS V2.1 is not recommended.

In this study, prostate volume was measured using 3T MRI, and PSAD was then calculated. PSAD demonstrated outstanding diagnostic performance for PCa and CSPCa when utilizing the cut-off values of 0.38 and 0.65, with AUC values of 0.84 and 0.89, respectively. This study’s PSAD cutoff value was much higher than 0.15, which may be a result of the population’s generally high PSA levels (median 14.21 ng/ml). The PI-RADS v2.1score and PSAD were independent predictors of CSPCa, according to multivariate logistic regression analysis. According to a prior study, PSAD not only predicts the results of prostate biopsy but also is a predictor for CSPCa. Kosaka et al. reported that PSAD could become a useful predictor of significant PCa in men aged ≤ 50 (19). According to a number of studies, higher PSAD is an important independent predictor of pathological upgrade between prostate biopsy and radical prostatectomy (20–23), and PSAD derived from MRI shows a more significant correlation with CSPCa compared with using TRUS (24). So, PI-RADS v2.1 score and PSAD were applied as risk factors in the prediction models for CSPCa. We reported that the diagnostic performance of the model was significantly better than each single variable (p <0.05). Despite the paucity of studies employing PI-RADS v2.1 score combined PSAD, outcomes from studies employing PI-RADS v2.0 score combined PSAD have been inconsistent. Several studies have shown that PI-RADS v2.0 score combined with PSAD as a screening tool had a higher predictive value for CSPCa (11, 12, 21, 25). Using the PI-RADS v2.1 score combined with PSAD as a screening tool for CSPCa, our study demonstrated a better predictive effect. However, Cuocolo et al. found that PSAD combined PI-RADS v2.0 score did not show a significant improvement in the diagnostic performance (26).

In this study, if the PI-RADS v2.1 score of 3 was the recommended threshold for biopsy, 19.2% of patients would have received an unnecessary biopsy. The specificity was fair low. Although the calculators reported were useful for predicting CSPCa (27), they are not convenient and practical for clinicians. As a result, we divided PSAD into four subgroups based on the cut-off points for PCa and CSPCa identified in this study (0.38, 0.65) and accepted threshold (0.15), and then combined them with PI-RADS v2.1 scores to constructed the risk category of CSPCa. The absence of CSPCa in the low-risk group suggests that 44.8 percent (189/422) of patients might have avoided unnecessary biopsies, and CSPCa would not have been missed. Furthermore, for high-risk patients who got negative results in the first biopsy, risk stratification could help to formulate a follow-up strategy.

Washino’s research (10) had confirmed that a combination of PI-RADS v2 score and PSA density can assist with prostate biopsy decision-making. The most significant difference between our study and theirs was the replacement of PI-RADS 2.0 with PI-RADS V2.1. Correspondingly, the calculation method of prostate volume has also changed. As an improved version, PI-RADS V2.1 is more accurate than PI-RADS V2.0 in diagnosing CSPCa, according to our research. Moreover, there were less PI-RADS scores of 3 in our study than in Washino’s (42.0%, 122/20 vs. 12.0%, 53/422), which was regarded as the probability of CSPCa being uncertain, making its diagnosis extremely difficult. Second, compared to Washino’s research10, which merely classified PSAD subgroups based on a simple multiple relationship of 0.15ng/ml2, our study is more detailed. Our research established more subgroups and performed more thorough risk classification. Thirdly, our analysis comprised a bigger sample size and tracked individuals with a negative first biopsy for up to two years. Overall, our study was one of the few to evaluate the effectiveness of the combined PI-RADS V2.1 and PSAD scores in predicting biopsy outcomes. Our research not only supports prior findings but also serves as a foundation for future studies.

Additionally, certain studies are useful as clinical references. In patients with PSA levels between 4 and 10 ng/mL, the combination of PI-RADS v2.0 and PSAD has been demonstrated to improve the predictive value of CSPCa and reduce the number of unnecessary biopsies (28, 29). In addition, the combination also improves predictive value of CSPCa in targeted prostate biopsy and reduce unnecessary biopsies (12, 30, 31).

We did not include multiple CSPCa definitions in the meta-analysis due to the substantial variability in NPV that was caused by the various definitions. Consequently, this would have brought unacceptable clinical heterogeneity into the data, possibly leading to erroneous and biased estimations. Last but not least, various factors, such as racial differences radiologists’ experience (32), etc., influence the outcomes of different studies.

There are several limitations in this study that need to be noted. First of all, it is a retrospective single-center study, and prospective validation is lacking because of insufficient follow-up time. Second, although the previous study has shown that MRI/US cognitive fusion-targeted biopsies(COG-TB) are superior to systemic biopsies in detecting PCa (33), MRI/US COG-TB also exists false negative, which may result in possible bias (34, 35). Third, our outcomes were assigned according to biopsy-proven Gleason score and mpMRI-proven T staging, which deviates from the pathology results after radical prostatectomy.

## Conclusion

In the present study, PSAD and PI-RADS v2.1 scores demonstrated more predictive value than tPSA, f/tPSA, PV, and age. We utilized the PI-RADS v2.1 score and PSAD as jointed factors to diagnose PCa and CSPCa, which displayed significantly greater predictive value. In the risk category we constructed, patients with PI-RADS v2.1 score of ≤2, or PI-RADS v2.1 score of 3 and PSA density of <0.15 ng/mL^2^, could avoid unnecessary prostate biopsy without missing clinically significant prostate cancer. In conclusion, our study offers a novel predictive risk category to improve the diagnosis of CSPCa while preventing unnecessary biopsies for clinicians.

## Data availability statement

The original contributions presented in the study are included in the article/supplementary material. Further inquiries can be directed to the corresponding authors.

## Authors contributions

X-DL and JH designed the study and provided funding acquisition. YL, T-JL, and PG conducted the initial retrospective analysis, participated in the study, analyzed the data, and prepared the initial manuscript, collected clinical data and performed patients followed-up. Y-KY, CG, and LZ measured the prostate volume and calculated the PI-RADS v2.1 score. All authors contributed to the article and approved the submitted version.

## Funding

This study was supported by the National Natural Science Foundation of China (Grant No. 81802548, 81860451), Yunnan Health Training Project of High Level Talents (for Peng Gu, Grant No. H2018070), Provincial Natural Science Foundation of Yunnan-Kunming Medical University Joint Foundation (Grant No. 2019FE001-136), and Scientific Research Project of Yunnan Provincial Educational Department (Grant No. 2018JS208). Funding for young doctors (for Peng Gu), from the 1st Affiliated Hospital of Kunming Medical University (Grant No. 2017BS016). Supported by Priority Union Foundation of Yunnan Provincial Science and Technology Department and Kunming Medical University 2017FE467 (-136).

## Conflict of interest

The authors declare that the research was conducted in the absence of any commercial or financial relationships that could be construed as a potential conflict of interest.

## Publisher’s note

All claims expressed in this article are solely those of the authors and do not necessarily represent those of their affiliated organizations, or those of the publisher, the editors and the reviewers. Any product that may be evaluated in this article, or claim that may be made by its manufacturer, is not guaranteed or endorsed by the publisher.
